# Evaluation of State-Led Surveillance of Neonatal Abstinence Syndrome — Six U.S. States, 2018–2021

**DOI:** 10.15585/mmwr.mm7102a1

**Published:** 2022-01-14

**Authors:** Shahla M. Jilani, Kristina West, Laura Jacobus-Kantor, Mir M. Ali, Alice Nyakeriga, Heather Lake-Burger, Meagan Robinson, A. Elise Barnes, Tracey Jewell, Shayne Gallaway

**Affiliations:** ^1^Office of the Assistant Secretary for Health, U.S. Department of Health and Human Services, Washington, DC; ^2^Office of the Assistant Secretary for Planning and Evaluation, U.S. Department of Health and Humans Services, Washington, DC; ^3^Tennessee State Department of Health; ^4^Florida Department of Health; ^5^Virginia Department of Health; ^6^Georgia Department of Public Health; ^7^Kentucky Department for Public Health; ^8^Arizona Department of Health Services; ^9^Division of Population Health, National Center for Chronic Disease Prevention and Health Promotion, CDC.

Opioid use disorder (OUD) is a significant public health problem in the United States, which affects children as well as adults. During 2010–2017, maternal opioid-related diagnoses increased approximately 130%, from 3.5 to 8.2 per 1,000 hospital deliveries, and neonatal abstinence syndrome (NAS) increased 83%, from 4.0 to 7.3 per 1,000 hospital deliveries (*1*). NAS, a withdrawal syndrome, can occur among infants following in utero exposure to opioids and other psychotropic substances (*2*). In 2018, a study of six states with mandated NAS case reporting for public health surveillance (2013–2017) found that mandated reporting helped quantify NAS incidence and guide programs and services (*3*). To review surveillance features and programmatic development in the same six states, a questionnaire and interview with state health department officials on postimplementation efforts were developed and implemented in 2021. All states reported ongoing challenges with initial case reporting, limited capacity to track social and developmental outcomes, and no requirement for long-term follow-up in state-mandated case reporting; only one state instituted health-related outcomes monitoring. The primary surveillance barrier beyond initial case reporting was lack of infrastructure. To serve identified needs of opioid- or other substance-exposed mother-infant dyads, state health departments reported programmatic successes expanding education and access to maternal medication for opioid use disorder (MOUD), community and provider education or support services, and partnerships with perinatal quality collaboratives. Development of additional infrastructure is needed for states aiming to advance NAS surveillance beyond initial case reporting.

A 2018 study (*3*) identified six states (Arizona, Florida, Georgia, Kentucky, Tennessee, and Virginia) with laws mandating NAS case reporting by applying specific criteria focusing on laws across all 50 states and the District of Columbia that explicitly named “neonatal abstinence syndrome” in disease and conditions reporting laws. Although each state reported distinct pathways for law enactment, state officials consistently indicated that the purpose for mandating NAS reporting was to characterize both NAS incidence and impact in the state and to identify more severely affected communities and opportunities for programmatic development. One of the main findings from that study indicated that mandated reporting helped quantify NAS incidence and guide programs and services. Accordingly, the overarching aim of the current study was to review surveillance features and program development by the same six states, after the enactment of state laws, to better understand NAS surveillance beyond initial case reporting. Thus, this qualitative study was designed to examine longer-term surveillance and programs developed postimplementation as a primary objective, and changes since the 2018 study in data collection and quality assurance practices as a secondary objective. Epidemiologists and birth defects program managers from all six states completed the 34-item questionnaire and semistructured follow-up telephone interview during February–April 2021. Questionnaire and interview data were analyzed for similarities and differences in initial case reporting (timeliness, reporting criteria, and completeness) and features beyond initial case reporting (outcomes follow-up, quality assurance measures, and resources used) and, although not directly linked to surveillance programs, subsequent programmatic development since enactment of state-mandated NAS case reporting. This activity was reviewed by CDC and was conducted consistent with applicable federal law and CDC policy.*

A review of the programs indicated both differences and similarities across the six states’ surveillance features (Table 1). Important distinctions centered around data timeliness, with some additional variation in state-specified reporting criteria, and the least amount of variance in case follow-up and in use of reports. Case reporting typically occurs within 30 to 66 days in Georgia, Kentucky, and Virginia, with the shortest reporting time noted by Tennessee (28 days) and the longest by Florida (180 days). As in the 2018 study, all six states reported that clinical diagnosis, regardless of whether treatment was given, prompted NAS case reporting (reporting varies from state to state). However, both Georgia and Tennessee reported transitioning to implementation of the NAS case definition recommended by the Council of State and Territorial Epidemiologists (CSTE) to standardize use in provider reporting with clinical record documentation and administrative claims-based data (*4*). Most states estimated receiving reports for approximately 75% of total NAS cases diagnosed by clinicians; Arizona receives reports for 50% to 75% of total cases. Consistent with the 2018 study, states collectively use case reporting to determine 1) incidence of NAS, 2) substance exposure patterns within different geographic and demographic communities, and 3) program development within the respective states. Kentucky also uses case reporting to characterize hospital discharge disposition for mother and infant.

**TABLE 1:** Features of neonatal abstinence syndrome case reporting — six states,* 2018–2021^†^

State (yr)^§^	Reporting timeliness,^¶^ days	Reporting criteria: clinician diagnosis**	Case follow-up^††^	Estimated completeness of case capture,^§§^ %	Use of case reports	
					To determine NAS incidence, community substance use patterns, and guide program development	To characterize mother-infant hospital discharge disposition
Arizona (2017)	Unknown**^¶¶^**	Yes	None	50–75	Yes	No
Florida (2014)	180	Yes	None	>75	Yes	No
Georgia (2017)	51	Yes: infant toxicology positive.*** transitioning to CSTE case reporting definition.^†††^	None	>75	Yes	No
Kentucky (2014)	66	Yes	None	>75	Yes	Yes
Tennessee (2017)	28	Yes: transitioning to CSTE case reporting definition.^†††^	None	>75	Yes	No
Virginia (2017)	30	Yes	None	>75	Yes	No

**Abbreviations:** CSTE = Council of State and Territorial Epidemiologists; NAS = neonatal abstinence syndrome.

* Arizona, Florida, Georgia, Kentucky, Tennessee, and Virginia.

^†^ The six states that implemented mandatory NAS reporting during 2013–2017 were invited for voluntary participation in a follow-up questionnaire and telephone interview to review NAS case reporting and surveillance from May 2018 to February 2021.

^§^ Year legal NAS case reporting mandate became effective; Florida had passive NAS case reporting system from the Agency for Health Care Administration within 6 months of diagnosis.

^¶^ Average number of days from the time of NAS diagnosis to case report.

** Medical provider diagnosis regardless of whether infant required or was given specific treatment.

**^††^** System or standard operating procedure in place for follow-up of infants with diagnosed NAS or their families once state health department has been notified of the case.

^§§^ Capture of total case incidence rate via case reporting compared with hospital discharge records.

**^¶¶^** Timeliness is unknown because state-level resources to analyze and monitor completeness received limited NAS case reports.

*** For Georgia, infants with positive toxicology or clinician diagnosis of NAS are reported.

^†††^ https://cdn.ymaws.com/www.cste.org/resource/resmgr/2019ps/final/19-MCH-01_NAS_final_7.31.19.pdf

Alongside information on surveillance extending beyond initial case reporting, states also noted numerous ongoing case reporting challenges (Table 2). These include collecting missing information (e.g., race or ethnicity, toxicology data, and substance exposure history) for mother or infant; assessing data accuracy from electronic health records, claims data, and medical record abstraction; sharing reports with other agencies; and de-duplicating data received from multiple sources. To reduce missing data, Kentucky and Tennessee instituted mandatory data fields and linkage of case reporting to vital records. Georgia noted that providing reporter education on case reporting best practices and partnering with national laboratories for electronic reporting of positive infant toxicology were helpful to initial case reporting efforts.

**TABLE 2:** Features of state-led surveillance of neonatal abstinence syndrome in states with mandated reporting* — six states, 2018–2021

Program feature	Surveillance findings reported by health officials^†^	States implementing surveillance feature
Ongoing challenges with initial case reporting^§^		
Resource-intensive activities (surveillance-related activities requiring the most state resources)	Collecting missing information (infant)	Arizona, Georgia, Tennessee, Virginia
	Collecting missing information (mother)	Arizona, Georgia, Tennessee, Virginia
	Assessing data accuracy (medical record abstraction)	Florida
	Sharing reports with local, state, and federal agencies	Tennessee
	Deduplicating data received from multiple facilities and medical providers	Georgia, Kentucky, Virginia
	Tracking and reconnecting with families of infants relocating within state	Arizona, Virginia
Barriers to initial case reporting	Lack of capacity to carry out medical record abstractions	Tennessee
	Limited awareness of surveillance efforts by facilities, medical providers, or staff turnover	Georgia, Kentucky
	Variability in case identification and reporting by facility	Georgia
	Passive surveillance registry limits timeliness, accuracy, and data completeness	Florida
	Challenges with criteria or implementation of NAS case definition	Arizona, Georgia
**Activities beyond initial case reporting^†^**		
Health-related outcomes^¶^ (e.g., maternal OUD or SUD, initiation or retention in MOUD program, infant hospitalization rates and comorbidities)	Monitoring comorbidities in infants with NAS	Kentucky
	Monitoring infant hospitalization rates	Kentucky
	Monitoring rates of infant preventative health maintenance visit, vaccine information	Kentucky
Social services-related outcomes^¶^ (e.g., linkage to housing, transportation, food or nutrition, child welfare, legal assistance, or juvenile courts services)	N/A	None
Development-related outcomes^¶^ (e.g., linkage or retention in Head Start, early intervention, home nursing visitation services)	N/A	None
Program development or improvement activities informed by state NAS surveillance** (to serve identified needs of opioid or substance-exposed mother-infant dyads)	OUD education campaign (e.g., stigma reduction) for providers and families	Arizona, Kentucky, Tennessee
	Expand MOUD programs for pregnant or postpartum women	Arizona, Florida
	Educational outreach to local MOUD providers and jails for expanded access to contraception for persons voluntarily seeking contraception	Tennessee
	Educational or training outreach to hospitals participating in quality improvement program initiative to improve care management for NAS	Georgia
	Teleconsultation program for providers on maternal substance use prevention and treatment	Virginia
	Plan of Safe Care program designed specifically to identify OUD in pregnancy and link to MOUD	Florida
	Expand reimbursement for OUD screening or intervention	Florida
Policy enactment informed by state NAS surveillance** (to address needs of opioid or substance-exposed mother-infant dyads)	Broadened same-day long-term contraception availability through state Medicaid program	Tennessee
Barriers to follow-up of initial case reports	Lack of infrastructure within agency to conduct follow-up with families of infants with reported cases of NAS	Arizona, Florida, Georgia, Tennessee, Virginia
	Lack of infrastructure at outside agencies that provide services to families of infants	Arizona, Virginia
	Lack of access to necessary infrastructure or services in rural communities	Kentucky, Tennessee
**Quality assurance measures and resources as reported by health officials^§,††^**		
Institution of required data fields	+ Collecting missing data	Kentucky, Tennessee
Link case report data to vital records	+ Collecting missing data	Kentucky, Tennessee
Health official review of reported cases	- Requiring more resources to carry out activity	Kentucky, Tennessee
Request additional or missing information	- Collecting missing data; burdensome, inefficient	Georgia, Tennessee
Reporter education on best practices to complete case report	+ Collecting missing data and data quality	Georgia, Tennessee
Partnering with national laboratories to receive positive toxicology for infant via ELR	+ Enabling confirmation of select reported results and identification of cases that may have been otherwise missed	Georgia
	- Laborious to set up	
Tools or resources used (local or community or state-level resources used in conducting surveillance)	+ Partnering with reporting hospital staff	Georgia, Tennessee
	+ Using web-based electronic reporting tools	Georgia, Kentucky, Tennessee
	- Faxing reports	Kentucky
	+ Partnering with state perinatal quality collaborative	Florida, Georgia, Kentucky, Tennessee, Virginia
	+ Using existing state disease reporting system streamlines hospital reporting	Arizona
	+ State mandate for NAS public health reporting	Arizona, Georgia, Tennessee, Virginia

**Abbreviations:** ELR = electronic laboratory reporting; MOUD = medication for opioid use disorder; NAS = neonatal abstinence syndrome; OUD = opioid use disorder; SUD = substance use disorder; + = most helpful; − = least helpful.

* Arizona, Florida, Georgia, Kentucky, Tennessee, and Virginia.

^†^ Surveillance findings listed are summarized from responses to questionnaires and semistructured interviews completed by state health departments.

^§^ Including and extending beyond initial case reporting; surveillance features listed are summarized from question items detailed in both questionnaire and semistructured interview completed by state health departments.

^¶^ Monitoring of specified outcomes since enactment of state-mandated NAS case reporting.

** Programs developed or policies enacted since institution of state-mandated NAS case reporting.

^††^ Quality assurance measures enacted to improve completeness of case reporting.

States were asked about resources most and least helpful to surveillance efforts. Georgia and Tennessee noted that partnership with reporting hospital personnel and the use of free web-based reporting tools were helpful. Arizona officials noted that using an existing state disease reporting system streamlined hospital-based case reporting but noted that their state’s NAS case definition only accounted for opioids. Georgia reported that even after transitioning to the CSTE case definition, opportunities for improvement remain, including case definition implementation by medical provider and facilities to continue standardizing reporting using clinical and administrative data sources. Florida, Georgia, Kentucky, Tennessee, and Virginia reported that partnering with perinatal quality collaboratives was helpful for ongoing surveillance efforts offering improvement opportunities for 1) case reporting, data collection, and data quality; 2) clinician education on resources for opioid and substance-exposed infants and mothers, and 3) health outcomes tracking.

States were also asked about monitoring health-, social services-, and developmental-related outcomes. No states reported available capacity to follow up on use of social services or developmental-related outcomes. With the exception of Kentucky, states reported that they did not monitor health-related outcomes. Kentucky has instituted state-level monitoring of infant hospitalization and comorbidity rates, and preventive health maintenance and vaccination rates, facilitated by direct linkage and data-sharing with their state Medicaid program. Overall, officials reported a lack of infrastructure (personnel, resources, and data linkages) within state health departments and outside agencies as primary reasons for limited long-term surveillance of NAS. Florida described their passive case reporting system as limiting timeliness, accuracy, and data completeness, and consequently, affecting downstream follow-up and surveillance.

## Discussion

The current study was designed to review NAS surveillance beyond initial case reporting and program development after implementation of state-mandated NAS case reporting; however, none of the six states report follow-up of infants or families beyond the initial NAS case report. Notably, initial state reporting mandates were intended to improve short-term timeliness of NAS epidemiologic data collection, not necessarily long-term follow-up or surveillance. Consequently, most reporting programs were not initially linked to existing health, social services, or developmental follow-up programs within states, explaining the significant data-sharing gap. Only one state has been able to monitor infant health-related outcomes and, despite ongoing interest in long-term outcomes, none of the six states has been able to track use of social services or development-related outcomes. The states cited critical infrastructure gaps as limiting their ability to conduct longer-term surveillance and reported distinct care access gaps in rural communities (e.g., geographic and internet bandwidth limitations). This limitation is concerning given 2004–2017 data showing disparities in OUD and NAS incidence across rural versus urban regions and remote rural counties (*1*,*5*,*6*). Considerations for infrastructure development to support long-term surveillance include capacity-building measures for 1) sufficient personnel (e.g., epidemiologist, data or information technology manager, or developmental specialist), 2) technical architecture (electronic system for housing longer-term surveillance data or data linkages to other state systems), and 3) legislative pathways to address potential confidentiality barriers regarding data-sharing between state health departments and other state agencies.

Despite state health department-reported infrastructural limitations in surveillance beyond initial case reporting, the six states with mandated NAS reporting have been able to achieve several noteworthy programmatic developments to serve identified needs of opioid and substance-exposed mother-infant dyads. Many of these developments focus on educational programs for medical providers and families to serve identified needs of opioid and substance-exposed infants and families, including stigma reduction (Arizona, Kentucky, and Tennessee). Georgia has implemented a quality improvement initiative centering around hospital educational outreach to improve NAS care management. Tennessee conducted educational outreach to providers of MOUD and local jails to expand access to contraception for persons voluntarily seeking access. Virginia provides educational teleconsultation to medical providers on OUD prevention and treatment. Florida is in the process of applying the Plan of Safe Care model for infants and families to a parallel program identifying pregnant women with OUD to link to MOUD, essential for a mother-infant dyad care model (*7*,*8*). With respect to policy enactment, Tennessee has broadened voluntary long-acting reversible contraception availability through Medicaid, a policy partially informed by state-mandated NAS case reporting.

The findings in this report are subject to at least two limitations. First, because this analysis relies largely on qualitative data, it cannot quantify the impact of NAS surveillance in the six states. Second, this study is a follow-up of six states with mandated NAS case reporting implemented during 2013–2017; other states with reporting statutes and regulations not meeting the search criteria from the 2018 study are not included (*9*). As such, study findings from these six states might not be generalizable.

Although mandated NAS case reporting offers opportunities for short-term epidemiologic data collection, continued case reporting and infrastructural challenges limit the breadth of short- and long-term surveillance. With resource- and capacity-building assessments and responding actions, state health departments might be better prepared to bridge the gap between initial case reporting and longer-term needs analysis and support for affected infants and families.

**Summary**

**What is already known about this topic?**

Increasing diagnoses of maternal opioid use disorder and neonatal abstinence syndrome (NAS) continue to affect U.S. communities. During 2018, a study of six states with mandated NAS case reporting for public health surveillance (2013–2017) found that mandated reporting helped quantify NAS incidence and inform programs and services.

**What is added by this report?**

A follow-up study of these states found continued advantages in determining NAS incidence and community exposure patterns to guide state program development. However, persistent data collection challenges and infrastructural gaps influence states’ capacity for longer-term surveillance beyond initial case reporting.

**What are the implications for public health practice?**

States considering surveillance beyond initial case reporting might benefit from understanding opportunities and challenges related to necessary infrastructure and resource development to facilitate longer-term public health follow-up.

## Footnotes

* 45 C.F.R. part 46, 21 C.F.R. part 56; 42 U.S.C. Sect. 241(d); 5 U.S.C. Sect. 552a; 44 U.S.C. Sect. 3501 et seq.
